# Colossal left ventricular apical thrombus

**DOI:** 10.1002/ccr3.2081

**Published:** 2019-03-03

**Authors:** Judy Luu, Edney Boston‐Griffiths, Antonia Zhu, Davinder S. Jassal, Kunal Minhas

**Affiliations:** ^1^ Section of Cardiology, Department of Internal Medicine, Max Rady College of Medicine, Rady Faculty of Health Sciences University of Manitoba Winnipeg Manitoba Canada; ^2^ Department of Radiology, Max Rady College of Medicine, Rady Faculty of Health Sciences University of Manitoba Winnipeg Manitoba Canada

**Keywords:** cardiac mass, cardiac MRI, echocardiography, myocardial infarction

## Abstract

Left ventricular apical thrombus is a known complication following an anterior ST‐elevation myocardial infarction. Although left ventriculography may suggest an apical thrombus in the presence of a filling defect, additional imaging with echocardiography and/or cardiac magnetic resonance is strongly recommended to further characterize the thrombus post myocardial infarction.

## 1 CLINICAL IMAGES

**Figure 1:**
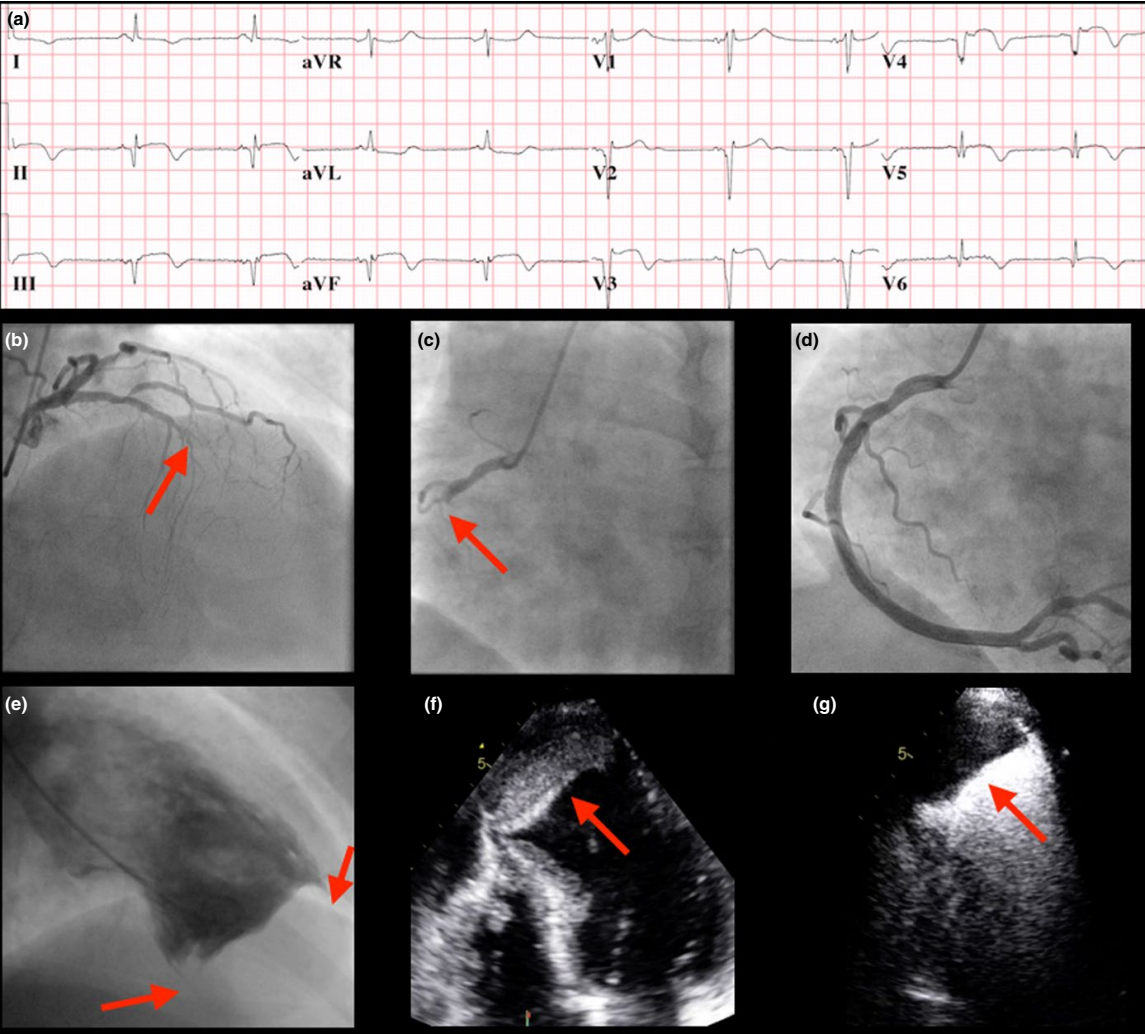
A, A 12 lead EKG on presentation demonstrating diffuse ST elevation and Q waves across the anterior and inferior leads. B, Coronary angiography of the mid left anterior descending artery (LAD) in the right anterior oblique (RAO) view demonstrating a chronic total occlusion (CTO) in its mid and distal segments. C, Coronary angiography of the culprit right coronary artery (RCA) in the left anterior oblique view demonstrating acute occlusion in its proximal segment. D, PCI of the RCA with implantation of a single drug‐eluting stent. E, Left ventriculography in the RAO view confirming an aneurysmal left ventricular (LV) apex with a large filling defect consistent with a thrombus. F, Apical 4 chamber view on transthoracic echocardiography (TTE) demonstrating a large layered thrombus (6.5 × 3.1 cm) at the LV apex that is aneurysmal and dyskinetic. G, Apical 4 chamber view on TTE confirming the large apical thrombus with no enhancement following the administration of Definity (Supporting information Videos [Link], [Link], [Link], [Link], [Link], [Link]B‐G)

A 57‐year‐old male presented with chest discomfort and an ECG demonstrating diffuse ST elevation in the anterolateral and inferior leads with Q waves (Figure 1A). Although the patient reported a similar episode of chest discomfort 8 months prior, he did not seek medical attention. Coronary angiography revealed a chronic total occlusion of the left anterior descending artery and acute occlusion of the dominant right coronary artery; the latter was revascularized (Figure [Link], [Link], [Link]B‐D). Left ventriculography revealed an akinetic apex with a large filling defect involving the distal half of the left ventricular (LV) cavity (Figure S1E). As transthoracic echocardiography (TTE) confirmed a large layered apical thrombus (Figure S1F‐G), the patient was anticoagulated with warfarin for 6 months. The incidence of LV thrombus in the percutaneous coronary intervention era is variable with a reported range of 15%‐25%.^1^, ^2^ Although left ventriculography may suggest an apical thrombus, echocardiography and/or cardiac magnetic resonance imaging are strongly recommended to further characterize the filling defect. Recommendations for the duration of systemic anticoagulation to prevent thromboembolic events is between 3 and 6 months.^1^, ^2^ Currently, there are no randomized controlled trials addressing the optimal treatment strategies or duration for LV thrombus post‐MI,^1^ and certainly not for one of this colossal size.

## CONFLICT OF INTEREST

None declared.

## AUTHOR CONTRIBUTION

JL, EBG, AZ, DSJ, and KM: contributed to the writing and approved the final manuscript.
